# Assessing gender responsiveness of COVID-19 response plans for populations in conflict-affected humanitarian emergencies

**DOI:** 10.1186/s13031-022-00435-3

**Published:** 2022-02-14

**Authors:** Yara M. Asi, Priliantina Bebasari, Emily Hardy, Michelle Lokot, Kristen Meagher, Emilomo Ogbe, Ateeb Ahmad Parray, Vandana Sharma, Claire J. Standley, Luissa Vahedi

**Affiliations:** 1 School of Global Health Management and Informatics, University of Central Florida, Orlando, FL USA; 2 Gender Specialist and Consultant, Jakarta, Indonesia; 3 Walsh School of Foreign Service, Georgetown University, Washington, DC, USA; 4 Department of Health Services Research and Policy, Faculty of Public Health and Policy, London School of Hygiene and Tropical Medicine, London, UK; 5 Conflict and Health Research Group, King’s College London, London, UK; 6 AISE Consulting, Ghent, Belgium; 7 James P Grant School of Public Health, BRAC University, Dhaka, Bangladesh; 8 Department of Global Health and Population, Harvard T.H. Chan School of Public Health, Boston, MA USA; 9 Center for Global Health Science and Security, Georgetown University, Washington, DC, USA; 10 Brown School, Washington University in St. Louis, St. Louis, MO USA

**Keywords:** COVID-19, Gender, Pandemic planning, Humanitarian emergencies, Conflict, Refugees, WHO-GRAS

## Abstract

**Background:**

The COVID-19 pandemic has necessitated rapid development of preparedness and response plans to quell transmission and prevent illness across the world. Increasingly, there is an appreciation of the need to consider equity issues in the development and implementation of these plans, not least with respect to gender, given the demonstrated differences in the impacts both of the disease and of control measures on men, women, and non-binary individuals. Humanitarian crises, and particularly those resulting from conflict or violence, exacerbate pre-existing gender inequality and discrimination. To this end, there is a particularly urgent need to assess the extent to which COVID-19 response plans, as developed for conflict-affected states and forcibly displaced populations, are gender responsive.

**Methods:**

Using a multi-step selection process, we identified and analyzed 30 plans from states affected by conflict and those hosting forcibly displaced refugees and utilized an adapted version of the World Health Organization’s Gender Responsive Assessment Scale (WHO-GRAS) to determine whether existing COVID-19 response plans were gender-negative, gender-blind, gender-sensitive, or gender-transformative.

**Results:**

We find that although few plans were gender-blind and none were gender-negative, no plans were gender-transformative. Most gender-sensitive plans only discuss issues specifically related to women (such as gender-based violence and reproductive health) rather than mainstream gender considerations throughout all sectors of policy planning.

**Conclusions:**

Despite overwhelming evidence about the importance of intentionally embedding gender considerations into the COVID-19 planning and response, none of the plans reviewed in this study were classified as ‘gender transformative.’ We use these results to make specific recommendations for how infectious disease control efforts, for COVID-19 and beyond, can better integrate gender considerations in humanitarian settings, and particularly those affected by violence or conflict.

**Supplementary Information:**

The online version contains supplementary material available at 10.1186/s13031-022-00435-3.

## Introduction

The COVID-19 pandemic and its subsequent social, health, and economic consequences have necessitated planning at all levels: local, state, and global. Planning allows for meaningful policies and guidelines that maximize benefits, minimize obstacles, and streamline costs [1]. In a public health crisis, planning allows states to predict and respond to the multitude of subsequent crises that stem from the original threat to health. In sectors as diverse as public transportation, education, and both local and state economies, response plans help actors respond to population needs and more quickly enter a post-crisis period [2].

The need to ensure an appropriate pandemic response is especially vital among populations affected by humanitarian emergencies, faced with limited resources and the many challenges associated with active conflict and forced displacement. In 2021, according to United Nations estimates, 235 million people worldwide were estimated to need humanitarian assistance [3]. Displaced populations, including refugees, are particularly vulnerable to infectious conditions given crowded living conditions, limited access to healthcare, lack of safe water and sanitation and other factors including poor underlying health and nutritional status [4]. During the pandemic, there have been significant disruptions to humanitarian initiatives and aid delivery in these contexts, adding to their fragility [5]. These populations, specifically civilians living in conflict-affected states and refugees fleeing these settings, will require significant and transformative planning at all levels to recede to a pre-pandemic status, let alone emerge from the conflict and instability that limited their health care prior to the pandemic.

Alongside the focus on developing preparedness plans and prevention strategies, there has been significant advocacy from scholars, humanitarian and development actors and activists on the need to ensure that the pandemic response recognizes the specific gendered impacts of COVID-19 [6–8]. As defined by the World Health Organization, gender refers to “the characteristics of women, men, girls and boys that are socially constructed”, fully implicating the role of attitudes, behaviors, norms, and policies of societies that create and perpetuate gender inequality [9]. Numerous guidelines, reports and technical briefs outline the importance of considering a range of gender issues in planning across sectors [10–12]. Gender-responsive plans and budgets are poised to not only lead to quicker pandemic recovery in general but offer a unique opportunity for states to tackle some of the inherent gender inequalities in societies [13].

A gender-responsive approach to addressing COVID-19 is not just for the benefit of women, but for the communities where women live, work, and study [14]. Pandemic control plans that explicitly address gender considerations are better positioned to address infectious disease outbreaks while also supporting the equity, human potential, capital, and economic integration of gender minorities, their families, communities, and wider society [15–17]. In essence, gender responsiveness is not a luxury or a “nice to have” but rather an essential and integral component of a sound public health policy.

In this paper, we analyzed the extent to which gender considerations have been integrated into pandemic plans. We examined the world’s complex humanitarian emergencies and displacement-related humanitarian crises and utilized an adapted version of the World Health Organization’s Gender Responsive Assessment Scale (WHO-GRAS) to determine the degree to which existing COVID-19 plans are gender-responsive [18]. This included classifying the pandemic responses to COVID-19 in environments of humanitarian crisis, defined here as states with active conflict or states that host significant refugee populations, as: gender-unequal; gender-blind; gender-sensitive; gender-specific or gender-transformative. We used the results to make specific recommendations for how response efforts for COVID-19 and beyond can better integrate gender considerations in humanitarian settings, and particularly address the needs of women, girls, and gender minorities and vulnerable populations affected by humanitarian crises.

## Methodology

### Country selection

**Fig. 1:**
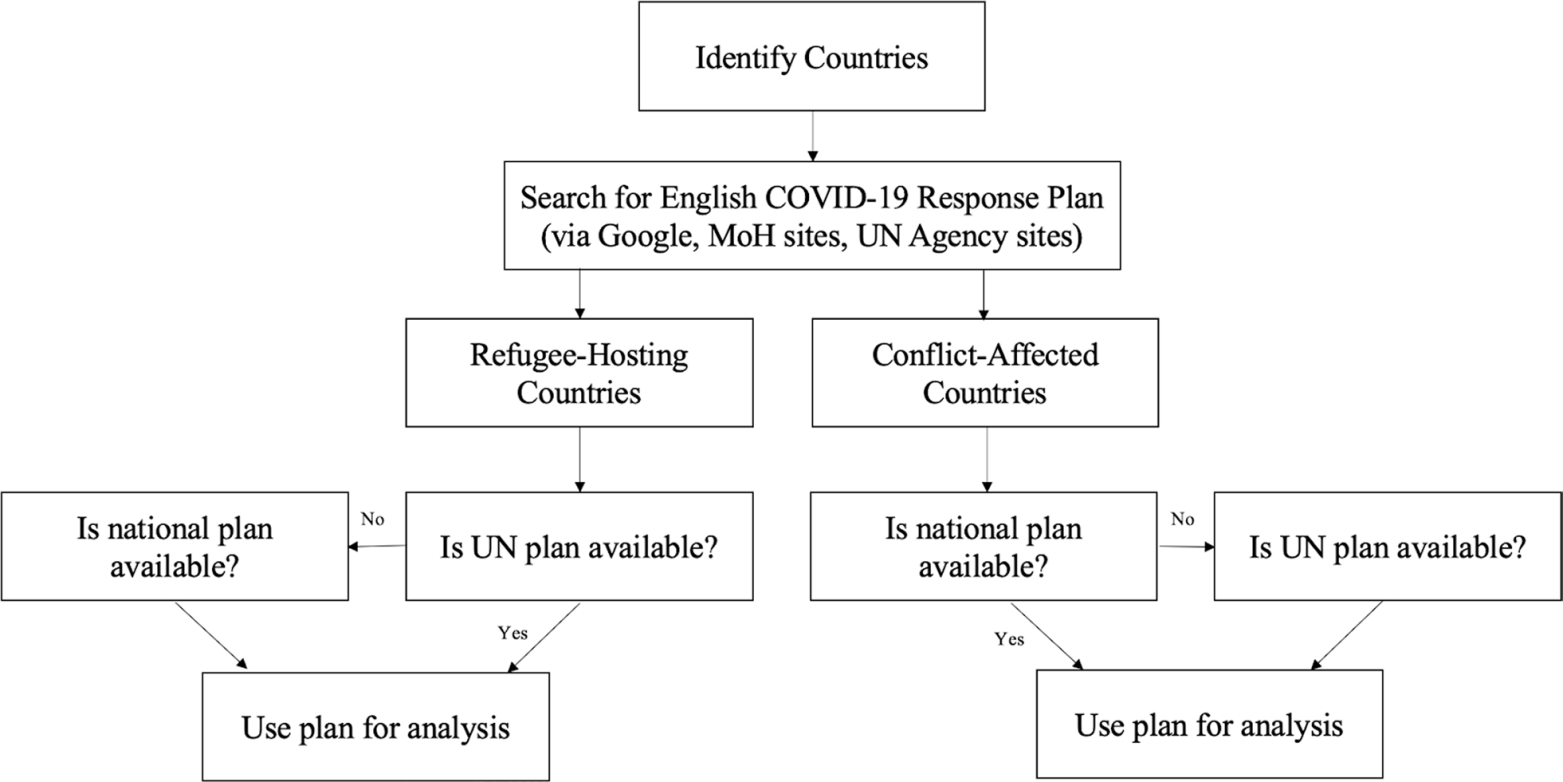
Identification and analysis process for COVID-19 response plans

**Fig. 2:**
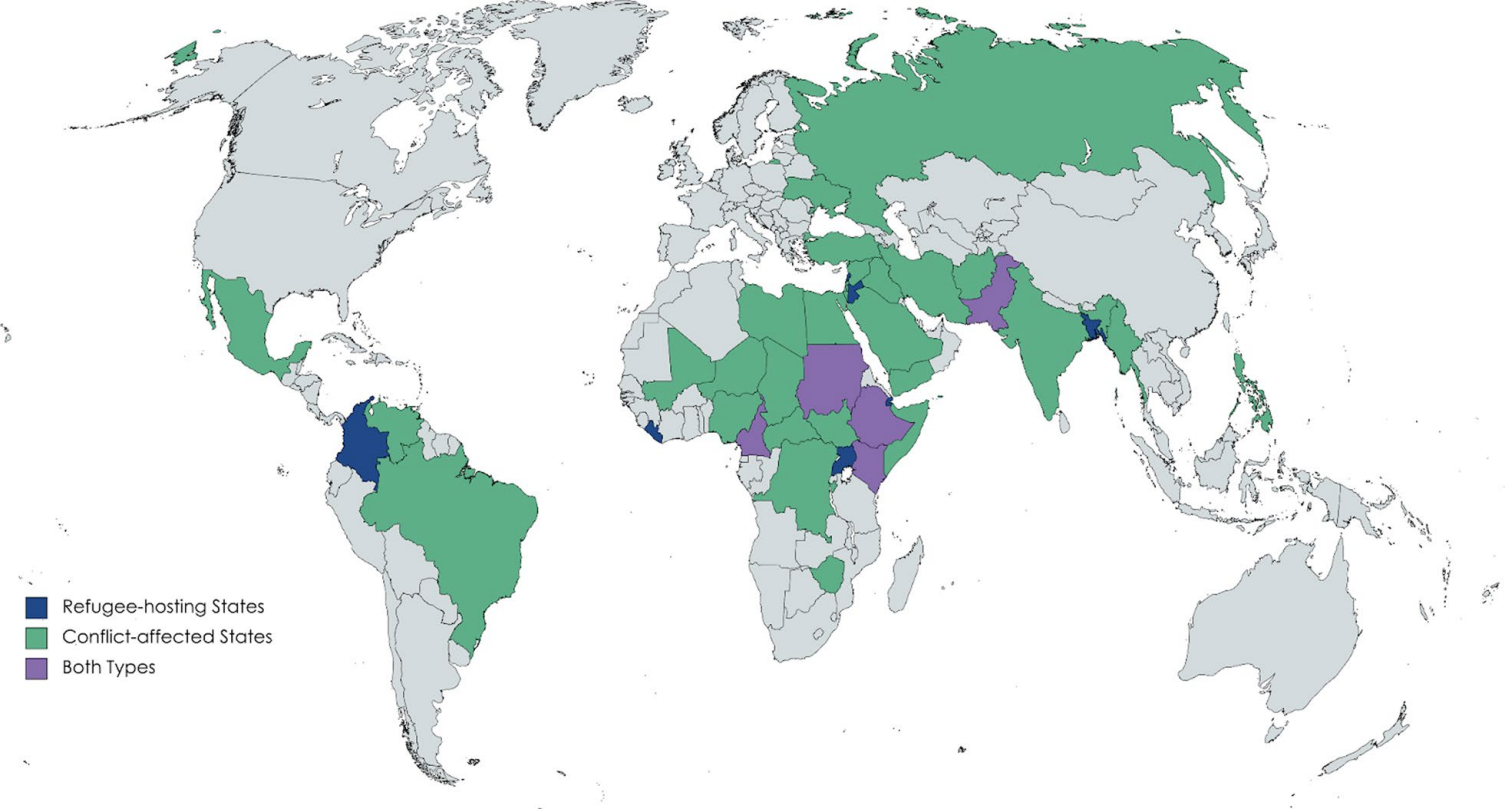
Map of refugee-hosting and conflict-affected states sampled for inclusion in study

We used a systematic multi-step process to identify the countries included in this analysis (Fig. 1). We divided countries into two groups: conflict-affected states and countries hosting forcibly displaced populations. For conflict-affected states, we targeted countries listed on the Uppsala Conflict Data Program (UCDP) database [19], which collects data about organized violence and conflict, from 2019 (the most recent year of data). Many of the states involved in some form of violent conflict are high-income states engaged in military efforts on foreign lands, and with largely insulated populations (such as the United Kingdom (UK) and Saudi Arabia). To ensure that the countries selected were directly conflict-affected, we cross-referenced the UCDP data with the 2020 Global Peace Index [20], which measures peace across three domains: *Safety and Security, Ongoing Domestic and International Conflict,* and *Militarization.* A lower ranking indicates a lower level of peace. We selected states with *low* or *very low peace* from the original UCDP list. Ultimately, 33 nations fit both criteria (Fig. 2; Table 1).

**Table 1:**
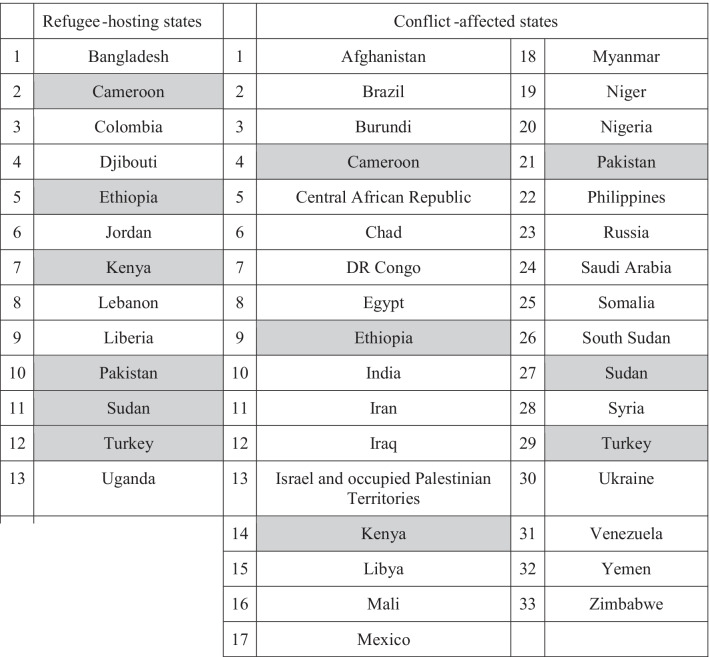
Refugee-hosting and conflict-affected states sampled for inclusion in study

Shaded box indicates a country on both lists

For the refugee populations, we used a similar two-step process. We selected the top refugee-hosting states as identified by the UN Refugee Agency (UNHCR) website [21]. We supplemented this list using the 2020 ranking of the top 10 refugee-receiving countries according to the Norwegian Refugee Council [22] an esteemed humanitarian agency that services refugees. After removing duplicates, we also excluded countries that were not directly neighboring the source of conflict from which the refugees were fleeing, or which were only receiving refugees through resettlement processes. This left a total of 13 countries (Table 1).

### Gender analysis

Drawing from Rosser et al., 2021 and UNICEF, 2020, we created a set of 16 binary (yes/no) indicators (Table 2). We classified the indicators into two primary groups: those that cover gender-specific topics (identified as gender-based violence; reproductive health; gender-disaggregated data collection; lesbian, gay, bisexual, transgender, queer or questioning, and other gender minorities [LGBTQI +] considerations, engagement with women’s groups, the gender digital divide) and those that are important for gender but are not specific to it (including disability, mental health, education, labor, caregiving, representation, and vaccination). A full description of each indicator and scoring approach is detailed in Additional file 1: File S1. Each plan was also designated as gender-unequal, gender-blind, gender-sensitive, gender-responsive, or gender-transformative, per WHO’s GRAS definitions [18]:

**Table 2:** List of indicators used in the study

#	Indicator	#	Indicator (cont.)
1	Gender-based violence (GBV)	9	Gender-sensitive data collection (including disaggregation)
2	Gendered domestic labor	10	Gender equality indicators for monitoring and evaluation
3	Gendered and unpaid caregiving	11	Mental health^a^
4	Gendered healthcare workforce	12	Gender representation in decision making, planning or implementation
5	Gendered impacts on income	13	LGBTQI + populations
6	Gendered impacts on education	14	Needs of persons living with disability(ies)^a^
7	Sexual/reproductive health services^a^	15	Gender in relation to Covid-19 vaccination
8	Engagement with women’s/girls networks or feminist groups	16	Gender digital divide

^a^These two indicators were counted as “yes” irrespective of whether gender was considered, and any specific mention of gender in relation to or intersecting with the indicator was separately noted

*Gender-unequal* Perpetuates gender inequality by reinforcing unbalanced norms, roles and relationships; privileges men over women (or vice versa); often leads to one sex enjoying more rights or opportunities than the other.

*Gender-blind* Ignores gender norms, roles and relations; very often reinforces gender-based discrimination; ignores differences in opportunities and resource allocation for women and men; often constructed based on the principle of being “fair” by treating everyone the same.

*Gender-sensitive* Considers gender norms, roles and relations; does not address inequality generated by unequal norms, roles or relations; indicates gender awareness, although often no remedial action is developed.

*Gender-specific* Considers gender norms, roles and relations for women and men and how they affect access to and control over resources; considers women’s and men’s specific needs; intentionally targets and benefits a specific group of women or men to achieve certain policy or programme goals or meet certain needs; makes it easier for women and men to fulfil duties that are ascribed to them based on their gender roles.

*Gender-transformative* Considers gender norms, roles and relations for women and men and how they affect access to and control over resources; considers women’s and men’s specific needs; addresses the causes of gender-based health inequities; includes ways to transform harmful gender norms, roles and relations; objective is often to promote gender equality; includes strategies to foster progressive changes in power relationships between women and men.

To ensure consistency, each identified response plan was reviewed by two reviewers; in case of disagreement, a third reviewer was asked to review and arbitrate.

## Results

### Overall trends and findings

We identified a publicly available, English-language COVID-19 preparedness and/or response plan for all our target countries except for Brazil, Burundi, Central African Republic, Chad, Iraq, Jordan, Liberia, Mexico, Russia, Turkey, and Venezuela (Fig. 1). We analyzed a total of 30 plans, of which 17 were published by the UN and/or other international organizations and 13 were published by national governments of the target countries. A number of the national plans noted contributions from external agencies, notably the World Bank, but were classified as “national plans” if hosted on a national web domain or if a government ministry was listed as the author of the plan. Six countries were selected as being both refugee-hosting and directly affected by conflict, of which three plans were published by the UN/international organizations and four were published by the national government; Pakistan was the only country to have two plans analyzed, as the only country affected both by conflict and hosting refugees per our classification for which both a UN/international plan and a national government plan were available for analysis (Table 3). Overall, of the 10 plans analyzed for countries hosting refugees (including countries also considered to be affected by conflict), seven were published by the UN/international organizations and three were national plans. For the conflict-affected countries (including those also hosting refugees), 13 plans were published by the UN/international organizations and 13 by the national government.

**Table 3:** Summary of identified plans and gender score

Country	Type of plan analyzed (National, UN/International)	Type of setting (Conflict-affected and/or Refugee-hosting)	Date published (MM/YYYY)	Publishing organization or entity	Number of pages	Gender score
Afghanistan	UN/International	Conflict-affected	06/2020	OCHA	130	Gender-specific
Bangladesh	UN/International	Refugee-hosting	NA/2020	Strategic Executive Group	56	Gender-specific
Cameroon	UN/International	Conflict-affected and Refugee-hosting	07/2020	OCHA	94	Gender-specific
Colombia	National	Conflict-affected and Refugee-hosting	05/2020	Humanitarian Country Team/UN Country Team Colombia	6	Gender-sensitive
Djibouti	UN/International	Refugee-hosting	05/2020	UN Country Team Djibouti	49	Gender-sensitive
DR Congo	UN/International	Conflict-affected	04/2020	World Bank	51	Gender-sensitive
Egypt	UN/International	Conflict-affected	07/2020	UN	53	Gender-specific
Ethiopia	National	Conflict-affected and Refugee-hosting	06/2020	Ministry of Health Ethiopia; Ethiopia Public Health Institute	155	Gender-sensitive
India	National	Conflict-affected	01/2020	National Health Mission	38	Gender-sensitive
Iran	National	Conflict-affected	04/2020	Islamic Republic of Iran, WHO, World Bank	5	Gender-blind
Occupied Palestinian Territories	UN/International	Conflict-affected	06/2020	World Bank	12	Gender-sensitive
Kenya	National	Conflict-affected and Refugee-hosting	02/2020	Ministry of Public Health, Republic of Kenya	34	Gender-blind
Lebanon	UN/International	Refugee-hosting	10/2020	UNHCR	12	Gender-blind
Libya	UN/International	Conflict-affected	04/2020	Health Sector Libya	22	Gender-sensitive
Mali	UN/International	Conflict-affected	10/2020	World Bank	11	Gender-sensitive
Myanmar	National	Conflict-affected	05/2020	Ministry of Education, Myanmar	74	Gender-sensitive
Niger	UN/International	Conflict-affected	03/2021	UNICEF, IOM and UNDP	6	Gender-sensitive
Nigeria	National	Conflict-affected	N/A	National Primary Health Care Development Agency, Nigeria	70	Gender-blind
Pakistan	UN/International	Conflict-affected and Refugee-hosting	04/2020	OCHA	48	Gender-specific
Pakistan	National	Conflict-affected and Refugee-hosting	04/2020	Government of Pakistan	58	Gender-sensitive
Philippines	National	Conflict-affected	03/2020	Department of Health, Philippines	279	Gender-specific
Saudi Arabia	National	Conflict-affected	N/A	Ministry of Health, Kingdom of Saudi Arabia	N/A	Gender-blind
Somalia	National	Conflict-affected	03/2020	Ministry of Health & Human Services, Somalia	17	Gender-blind
South Sudan	National	Conflict-affected	06/2020	Ministry of Health, South Sudan	45	Gender-sensitive
Sudan	UN/International	Conflict-affected and Refugee-hosting	07/2020	Humanitarian Country Team/UN Country Team Sudan	12	Gender-sensitive
Syria	UN/International	Conflict-affected	12/2020	OCHA	114	Gender-sensitive
Uganda	UN/International	Refugee-hosting	08/2020	UNHCR	102	Gender-specific
Ukraine	UN/International	Conflict-affected	03/2020	OCHA	22	Gender-sensitive
Yemen	National	Conflict-affected	06/2020	Ministry of Education, Yemen	33	Gender-sensitive
Zimbabwe	UN/International	Conflict-affected	05/2020	Zimbabwe Education Cluster	36	Gender-sensitive

**Fig. 3:**
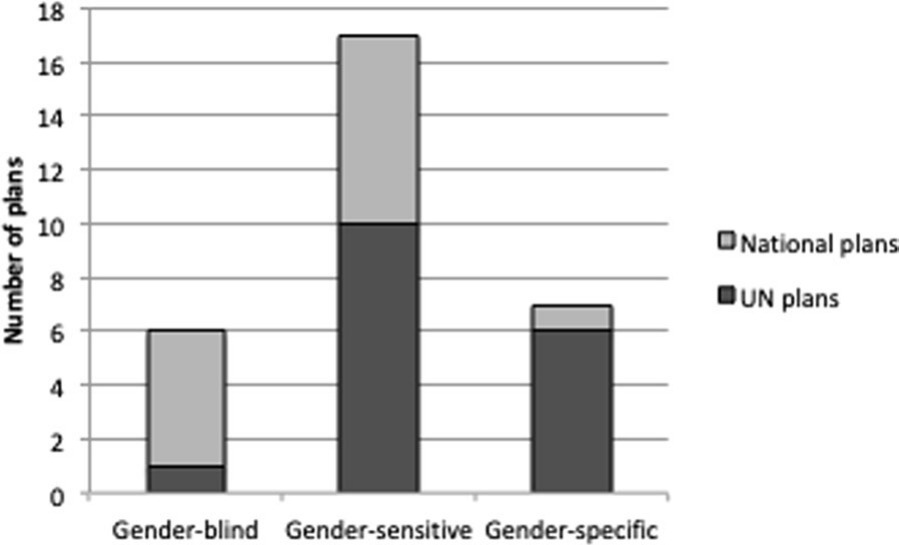
Summary of gender score across identified plans

Overall, six plans (20%) were scored as gender-blind, 17 (57%) as gender-sensitive, and seven (23%) as gender-specific (Fig. 3). None were scored as gender-unequal or gender-transformative. The five gender-blind national plans were from Iran, Kenya, Nigeria, Saudi Arabia, and Somalia. Of these, all were published by the Ministry of Health or a constituent agency, apart from Iran, in which a lead ministry was not specified. The one identified gender-blind UN/international plans was from Lebanon (UNHCR). The gender-specific national plan was from the Philippines, published by the Department of Health. The six gender-specific UN/international plans were from Afghanistan (OCHA), Bangladesh (focused on the Rohingya population, and published by the Strategic Executive Group), Cameroon (OHCA), Egypt (UN), Pakistan (OCHA), and Uganda (UNHCR).

### Gender indicators

The most commonly observed mention of gender in the plans was with respect to GBV, with 23 plans out of the 30 analyzed including it to some degree. There was strong recognition that the pandemic would have impacts on GBV across many plans, however not all the plans identified strategies to address GBV. Strategies that were identified included revising referral pathways for reporting (Djibouti), developing mass communication messages (Uganda, Sudan), and developing targeted prevention messaging for men (Bangladesh). In other cases, the strategy to address GBV appeared to be less concrete, for example stating the aim of reducing vulnerability to GBV (Kenya). The Ethiopia plan referenced ensuring “avoidance of any form of Sexual Exploitation and Abuse” through reliance on the WHO Code of Ethics and Professional conduct and ensuring segregated toilets and sufficient lighting in quarantine and isolation centers, which are aligned with global guidance on GBV risk mitigation during COVID-19 but did not include actions to address the norms and power hierarchies underlying sexual exploitation and abuse. The Uganda plan referred to the use of the ‘SASA!’ gender norms program (part of a community mobilization approach initially developed for preventing violence against women and HIV) [23] which pre-dated the pandemic, but it was unclear how this would be adapted or scaled-up to address violence during the pandemic. Moreover, in some countries (India, Philippines) plans mention the need to prevent sexual harassment among staff working in COVID-19 quarantine facilities.

The next most frequent context in which gender was explicitly mentioned was related to health care, and specifically access to sexual, reproductive or maternal health; almost two-thirds of plans (n = 19) included this content. Most of these plans, such as those of Colombia, Ethiopia, and India, mention reproductive health merely in the context of ensuring continuity of service, or having resources on hand to support the needs of pregnant women. The Sudan plan goes slightly further, acknowledging the secondary gendered impacts of lockdowns and curfews on women’s access to reproductive health services, but only includes child and maternal health under the “Maintaining essential health services and systems” pillar of preparedness and response activities. The Afghanistan plan does not address all aspects of reproductive health but does acknowledge the broader impacts of COVID-19 on women’s access to care: “*The combination of restrictions on men providing medical treatment to women and a shortage of women health professionals (particularly in rural areas) compromises women’s access to sustained and quality health care.”* To address these challenges, the plan includes an indicator related to the availability of female health workers at clinics, alongside others focused on ensuring continuity of and access to maternal health services.

Gender representation in leadership and decisions was identified in nine plans, and engagement with women’s/girl’s networks or related community groups in eight plans. The Afghanistan plan explicitly acknowledges the importance of greater inclusion of women’s voices in designing interventions:

*Gender imbalance in the humanitarian workforce is not only an issue at the point of aid delivery but also in coordination forums where it is critical that more women’s voices (particularly those of national female staff) are heard in discussions around programme design and protection risks.*

In other countries like Egypt, there was a focus on targeted stakeholder engagement of women and women groups, as well as a focus on activities to encourage women to engage in health governance activities, especially related to COVID-19. The Cameroon plan noted that “Attention will be paid to girls and women’s effective involvement in humanitarian decisions, considering the specific barriers they face to voice their concerns and be heard.” The Pakistan national plan specifically notes women’s groups as an example of a trusted community group under the "risk communication and community engagement" pillar; indeed, a priority sub-activity is to “Ensure community and women networks actively participate in awareness raising and community empowerment”. The Libyan and Philippines plans both have very similar language related to women’s groups as an example of a trusted community network. Of the plans analyzed, five discuss engagement of feminist advocacy groups or other gender activist entities. These include Afghanistan, Cameroon, Kenya, Iran, and Libya. Cameroon’s plan mentions engaging with existing women’s groups, civil society organizations and women’s rights organizations, given their ability to incorporate the voices of women and knowledge at all stages of the response.

Fewer than a quarter of the plans (n = 7) specifically referenced LGBTQI + populations, let alone their specific needs with respect to COVID-19. Those that did mostly discussed these populations and individuals in generic terms as a potentially vulnerable group, along with women, children, and people living with disabilities, who might require additional or targeted support. A few plans went further to discuss specific aspects of LGBTQI + populations’ needs. For example, the Uganda plan specifically mentioned LGBTQI + refugees from the Democratic Republic of Congo, related to low tolerance and acceptance among members of the public, although it did not go as far as to mention the legal context for why they might face discrimination in Uganda. The “Age, Gender and Diversity” approach is described as central to the Ugandan refugee response and requires that persons with diverse sexual orientations and gender identities and ethnicities are considered among other groups whose distinct needs and views must be integrated into assessment, planning, implementation and monitoring processes. The Pakistan national plan, under the “Case Management” pillar, notes the importance of mapping vulnerable populations and their ability to access care, with the “transgender community” specifically referenced. The Philippines national plan referenced “sexual and gender minorities” and specifically mentions “transgender people” as potentially at risk of social exclusion in the context of access to vaccination.

Gender-sensitive data collection was mentioned in 12 of the analyzed plans, almost exclusively in the context of collecting gender disaggregated data, although Egypt’s plan, developed by the UN with input from national partners, goes further by stating that “gender-responsive data” will be collected. Likewise, the Pakistan national plan lists the following as priority sub-activity under the country-level coordination, planning and monitoring pillar:

*Conduct a regularly updated, multi-sectoral gender analysis with sex, age and disability disaggregated data collection to identify inequalities, gaps, and capacities to assess the specific impacts of the crisis on the women, girls, men and boys of the affected population.*

Fewer plans specifically discuss gender equality indicators in the context of monitoring and evaluation of implementation of the plan, with only nine including such indicators. Most of these were focused on GBV; for example the DR Congo’s plan notes that GBV will be integrated into the overall monitoring and evaluation framework (although in the two brief monitoring and evaluation plans provided directly within the document, there is no mention of gender, let alone GBV). The Cameroon plan, published by OCHA, mentions Cameroon’s poor performance overall with respect to gender inequality indicators, and notes the importance of engaging women and girls in needs assessments, but does not actually include any specific gender-related objectives in the “Indicators and Targets” chapter. The Philippines plan covers both gender-responsive data collection and monitoring and evaluation through noting that “The M&E system will include data and information disaggregated by gender, demography, race-ethnicity, location-residence, socioeconomic status, and disability.”

Only three of the plans (Afghanistan, Cameroon and Egypt) discussed the digital gender divide. In Afghanistan, this was discussed with regards to fewer women having access to banking-enabled mobile phones, linked to barriers in having certain required forms of identification:

*Registered SIM cards are required to make full use of many mobile banking services and sign-up often requires the user to have a Tazkera or ID card. Few women have this ID, which creates an obstacle to independent use of mobile money.*

In Cameroon, the discussion was in the context of potential gender differences in access to and uptake of remote learning technologies and ensuring active participation of girls in these efforts, while in Egypt the response plan included a specific mention of “supporting the empowerment, livelihood and digital inclusion of rural women”.

#### General indicators with a gender component

Our analysis also included a number of indicators which at face value were not directly associated with gender but which are known to have, or could have, particularly strong gendered impacts.

Thirteen plans mentioned some aspect of unpaid caregiving, often gendered, and frequently in reference to the elevated risk of direct exposure to SARS-CoV-2, as well as other negative impacts. For example, the Libya plan notes that “Women caring for others, and the predominant role they play as health and social welfare responders, are particularly exposed to potential contamination.” The Ethiopia plan similarly highlighted risks faced by caregivers, and their need to be targeted for behavioral interventions and training but does not discuss gendered aspects of caregiving. The Syria plan mentions caregivers numerous times and integrates caregivers of young children with pregnant and lactating women under its maternal and child nutrition indicators, although these activities are not specifically in reference to the COVID-19 response.

A third of plans (n = 10) addressed domestic labor, with all acknowledging gendered roles. The Sudan plan in fact combined “domestic and caregiving burdens that women and girls perform” as activities associated with elevated health risks for women; the Djibouti plan had similar language, noting risk to women and girls was elevated due to their roles as “caretakers of the family,” and specifically citing caring for sick household members and fetching water as two tasks associated with this role. Focusing only on domestic labor, the Cameroon plan stated:

*Beyond the daily housework that weighs on women, the method of social distancing increases the family burden in terms of supplying and cooking food for the household and is a factor facilitating Gender Based Violence or Intimate Partner Violence.*

Woman and child-headed households are emphasized in the Syria plan as potentially particularly vulnerable to the impacts of lost education, employment, and health services caused by the on-going humanitarian crisis and conflict, which further touches on issues related to pandemic impacts on economy and income, mentioned in 11 of the plans. The Afghanistan plan, for example, also noted the different effects on female- and male-headed households, and the Djibouti plan mentions women heads of household as a vulnerable group. Several projects mentioned ensuring that implementation of social and economic protection aspects of the response plans will target women beneficiaries. For example, in the plan for the occupied Palestinian territories (West Bank and Gaza), at least fifty percent of beneficiaries under the “Cash for Work” (C4W) component must be women. The Sudan plan highlights the economic and employment impacts that measures used to curb SARS-CoV-2 transmission will have:

*Women and girls are more likely to experience a worsening of existing inequalities and disproportionate secondary impacts of restrictions to slow the pandemic as compared to men and boys. Curfews and lockdowns will limit their work and economic opportunities.*

A specific aspect of impact related to employment, as well as caregiving, involves the gendered nature of the health workforce in many countries, although this was only explicitly considered in seven plans. In the Djibouti plan, gender roles in the health workforce are acknowledged both in terms of direct provision of clinical care but also supporting roles:

*Women and girls are at heightened risk of exposure to the virus due to their common roles as front-line health workers or health facility service staff (e.g. cleaners, laundry etc.)*

The Philippines plan notes gendered aspects of the health workforce both in relation to the risk of GBV faced by women health workers, and also that health workers, “a big proportion who are female,” may be at substantial risk for mental health issues including burnout as a result of the pandemic. The Afghanistan plan takes a different tack, emphasizing the gender-responsive benefits that come with increased female participation in the health workforce, and directly encourages affirmative action policies with respect to recruiting women, tailored to the cultural context:

*While there is no overall census of female staff and volunteers working for humanitarian organisations in Afghanistan, women are, without doubt, grossly under-represented in the workforce. This remains a key constraint in terms of the response’s operational capacity to assess, understand and respond to the needs and concerns of women and girls. Measures are ongoing to redress this imbalance and recruit more women into humanitarian action include the hiring of husband-and-wife, as well as brother-and-sister teams. OCHA has employed additional female field monitoring staff for the AHF, while UNHAS offers reduced airfares for female national staff travelling on its flights as a way of encouraging managers to involve more women in field work, particularly assessments. A number of NGOs also have hiring policies for national staff that are designed to make it easier for women, who often have not had the same educational opportunities as men, to enter the humanitarian workforce.*

Ten plans mentioned some aspect of gendered impacts to education of the COVID-19 pandemic or response efforts. Three of the plans identified (Myanmar, Yemen and Zimbabwe) were specifically developed for the education sector, and all three made some mention of gender. Also referencing gendered aspects of household labor and economic impacts, the Myanmar plan noted that:

*Girls and female youth will also be more at risk of dropping out when education institutions reopen, due to the aggravation of the burden of domestic chores in the current context, but also to the risk of early marriage in poorest households, worsened by school closure.*

Non-education sector plans also mentioned impacts of COVID-19 on education. The Djibouti plan has several extensive sections on education, including several focused activities on strategies for ensuring educational continuity and access, with a brief mention that throughout such efforts, “12 *[sic]* minimize the increased risks in accessing services for this group.” Cameroon’s plan specifically mentioned gender with respect to education, as well as access to technology, linking back to the issue of the gender digital divide:

*Recognizing that there are gender disparities in access to technology and that literacy is gendered, boys being more often enrolled in primary and secondary schools than girls are, mixed methods that utilize multiple media options will be used. The Sector will ensure that girls and boys are equally involved in alternative, remote learning initiatives, while monitoring the regular participation of girls in these activities.*

Only one plan mentioned gender in relation to vaccination against COVID-19, although almost all of the analyzed plans were published prior to the approval of any vaccine candidates. The one plan that mentioned vaccination was the Philippines’, which notes that “sexual and gender minorities (especially transgender people)” could potentially be at risk of social exclusion, leading to reduced access to COVID-19 “information, treatment, and vaccines”.

Finally, we also considered two further indicators that are known to have close links with gender as well as intersectional impacts, to see if they were mentioned at all, and if yes, the extent to which any gender aspects were explicitly discussed. People living with disabilities (although the phrasing varied across plans) were mentioned in 17 plans, and mental health was included in 15 plans. However, fewer explicitly linked these issues to gender. The most common context in which gender was discussed alongside disability or mental health was with respect to sexual and gender-based violence. The Uganda plan, for example, notes that women and girls with disabilities may be at elevated risk of GBV. This was the only plan that explicitly linked gender and disability, although the Ethiopia plan also refers to protection measures against GBV in a section focused on mitigation measures for risks associated with disability, and the Afghanistan plan notes the need to improve analysis of the “gender, disability and mental health dimensions of the response”. With respect to mental health, gender was usually noted in terms of targeted provision of psychosocial support, again often related to sexual health or GBV. The Egypt plan, for example, described psychosocial support in relation to sexual and reproductive health services provided to women living with HIV, and the Niger plan lists community based mental health and psychosocial support for children, parents and primary caregivers under a child protection and GBV indicator. The Pakistan humanitarian response plan had a broader approach, acknowledging that overall anxiety and stress may be particularly acute for women and children during the response, “increasing the need for mental health and psychosocial services”.

### Intersectionality

Intersectionality recognizes the many ways in which other aspects of a woman’s identity, such as class and race, interact and overlap to produce the complex effects of oppressive and discriminatory structures [24]. While we did not initially seek to review the presence of intersectional language and policy, several plans recognized how power hierarchies and identities might intersect with gender to exacerbate inequalities that merit additional analysis. The Bangladesh plan, for example, observed how women’s lower educational status and role as primary caregivers increases their risk of contracting COVID-19; several other plans noted the overlay of infection risk related to gendered roles in households and the employment sector, and corresponding potential impacts on income, safety from violence, and access to education and health services. However, from the perspective of how intersecting identities can compound risk or vulnerability, it is worth noting that most plans listed these groups as discrete and separate, without recognizing overlapping categories. This is a major gap, especially in humanitarian contexts where displacement and stigma based on refugee status may add layers of discrimination on top of existing ones related to disability, gender, social status, ethnic group, religion, etc. Colombia’s plan provides one example:

*A large portion of women, children, disabled and homeless people, people with pre-existing health conditions, elderly, the LGBTI population as well as people belonging to indigenous communities, are particularly susceptible to disease due to social conditions both in terms of the impact of the disease, as well as the impact of the preventive measures.*

Another comes from the Afghanistan plan:

*Those who stand out as suffering the most are older people, people with co-morbidities, people with mental and physical disabilities, women, children and young people, IDPs, returnees, refugees and asylum seekers, and people who have lost their sources of income and fall outside social protection systems.*

Although elsewhere the Afghanistan plan does note the need for “better analysis of the gender, disability and mental health dimensions of the response,” these types of discrete lists were frequently observed in the response plans without acknowledgement that individuals could easily fall into two or more of the listed categories, with potential synergistic and exacerbated impacts.

However, a small number of plans did directly address intersectionality. The Uganda plan, for example, included this section in the “Age, Gender and Diversity and Accountability to Affected People” section:

*Forced displacement affect [sic] people differently, depending on age, gender and diversity. Understanding and analyzing the impact of intersecting personal characteristics on people’s experiences of forced displacement are necessary for an effective response.*

Likewise, the Yemen plan, which focused on the education sector, included a specific section on “Gender and Intersecting Inequities”, highlighting once again the importance of disaggregated data on not only understanding and addressing gender aspects of response planning, but where there may be intersecting impacts as well:

*Evidence has shown a weakness of data of most vulnerable groups, such as girls, children in remote areas, boys at risk of being recruited into armed groups/forces, children with disabilities, minorities and children out of school. The efforts will be put to produce qualitative reports to fill information gaps and commit to stronger disaggregation moving forward.*

## Discussion

One of the initial findings of our review of 30 COVID-19 response plans from conflict-affected humanitarian settings is that some countries do not have a unified pandemic response plan, or at least one that is publicly available. We also find gaps in how gender issues are considered in pandemic response plans. In total, 20% (n = 6) of all plans were classified as gender-blind, 57% (n = 17) were gender-sensitive and 23% (n = 7) were gender-specific. As our sample included a higher proportion of plans developed by UN/partner actors (57%, n = 17), who are often vocal in their promotion of gender equality issues, we anticipated greater content and analysis on gender. Our sample size of national plans was smaller (43.3%, n = 13) but what we observed was that national plans may be especially limited in how they consider gender issues. For example, Pakistan was the only country where both the UN plan and national plan were analyzed (as the country was both refugee-hosting and affected by conflict), and our findings indicate that the UN plan was classified as gender-specific and the national plan as gender-sensitive.

Despite overwhelming evidence about the importance of intentionally embedding gender considerations into the COVID-19 planning and response, none of the plans reviewed in this study were classified as ‘gender transformative’. While our study did not explore the reasons for this, others have drawn attention to problems with reducing consideration of gender to a tick-box exercise [25]. When gender is included to satisfy donor or organizational requirements, rather than being recognized as an important issue in its own right, the level of detail and responsiveness to gender considerations often tends to be limited.

In many of the plans we reviewed, there was acknowledgment of how gender relates to a particular issue, but then the plan did not go further to address the root causes of inequities or identify strategies to transform harmful gender norms, roles and relations. This indicates there is awareness of how gender relates to particular sectors, but less clarity on how to address gender inequality. The fact that GBV and reproductive/maternal health were the most common topics discussed in plans reinforces Percival’s (2018) critique that consideration of gender may be narrowly framed in terms of violence and maternal health [17], while other issues like women’s role in caregiving or in the labor force (particularly relevant for COVID-19) are less well recognized as they lack visibility. Lack of technical capacity or gaps in knowledge about strategies to promote gender equality may also have resulted in limited analysis beyond stating the gender inequality. It may be that more support is needed to translate identification of issues into tangible, and particularly transformative, interventions.

One specific area in which our analysis revealed opportunities for more concrete integration of gender into proposed interventions relates specifically to M&E indicators. While many plans contained rhetoric about the importance of gender, ultimately what gets measured gets done. There needs to be a much greater emphasis on developing and including gender-related indicators in M&E plans, and which go beyond GBV and reproductive health, for any hope of minimizing negative gendered impacts of the pandemic, let alone gender inequalities being meaningfully addressed. Moreover, plans may have to contain guidance on how to safely collect high quality, gender-sensitive data. The pandemic context poses challenges to primary data collection: stay-at-home orders may lead to privacy, confidentiality, and safety concerns regarding the collection of potentially sensitive data on GBV and sexual and reproductive health [24]. The use of technology (telephone, computers, smartphones, and mobile applications) to facilitate remote data collection is a promising practice, however, plans must outline best practices to utilizing such remote data collection tools.

While some plans mentioned LGBTQI + populations, this consideration did not tend to go beyond mentioning the vulnerabilities this group experiences. For example, in Uganda’s plan, LGBTQI + populations are discussed, however the plan does not propose interventions to engage in law reform or advocacy in context of Uganda’s criminalization of homosexuality [26].

Our analysis also highlights the untapped potential of partnering with local women’s groups and feminist activists. Women’s groups may be a particularly underutilized resource when it comes to advancing gender inclusion and transformation in refugee and conflict-affected settings. Their knowledge of their context and important role in communities is not always recognized, and yet may be particularly beneficial for ensuring response interventions are accepted, feasible, and sustainable. There is a need to engage not only women but also conflict-affected and refugee/IDP communities within decision-making, and potentially even co-production of response strategies [27], during pandemic planning so that their needs are reflected in response plans, another area that our analysis highlighted as a noticeable gap.

### Limitations

This paper has some limitations. Firstly, we were limited to analyzing COVID-19 plans that were accessible to the general public. Other COVID-19 plans that guide national decision making and policy which are not available to the general public may exist, however, they could not be included in our analysis. Furthermore, we specifically sought plans that were available in English, both for ease of analysis as well as to get a better understanding of the external-facing nature of these types of response plans. As our list of target countries was defined through use of existing databases, lists and indicators, it too is subject to certain limitations. For example, the most recent UCDP data was from 2019, meaning that more recent violent or conflict could have caused countries not to be included in that portion of the list. We also considered countries to be “conflict-affected” if UCDP identified any conflict or violence within the territorial borders, even though in some cases, violence or conflict might be geographically limited within a country, or to specific population groups. Similarly, as our search focused on countries hosting the most refugees worldwide, some countries that host smaller numbers of refugees would not have been classified as “refugee-hosting” in our analysis.

For the conflict-affected country list, if no national plans could be located or were unavailable in English, we then considered an agency plan from the UN or WHO that would apply in the respective country. For the refugee-hosting country list, we used agency plans from the UN or WHO at the first instance, then a government plan if agency plans were not available. However, we recognize that agency plans may not be appropriate proxies of COVID-19 planning and response within a given country. We utilized the WHO-GRAS to classify each located plan in relation to its consideration of gender [18]. Due to subjectivity in relation to applying the scale, we collectively interpreted any mention of women, girls, and gender as being gender-sensitive or above. We recognize that a mere mention of gender or related terms may not indicate a gender-sensitive policy approach, especially if the mention of gender is an afterthought. Finally, there is a difference between considering gender and related terms and outcomes in a policy document and implementing a gender informed pandemic control plan. Our analysis is limited only to what extent organizations and governments include and/or consider gender in their COVID-19 plans. Implementation of the plans with regard to gender is beyond the scope of this analysis.

### Recommendations

We suggest the following recommendations to ensure response plans for COVID-19 and any future pandemics/global crises meaningfully address gender inequalities. Many of these recommendations echo guidance and calls to action issued by other publications during the pandemic, however they clearly require reinforcement given the gaps identified during our analysis.

National governments should ensure that response plans:

Are evidence-based

- Are informed by sex (and age and disability) disaggregated data.
- Are developed by meaningfully integrating gender considerations and using gender analysis in their context.

Prioritize protection

- According to global GBV risk mitigation guidelines, address GBV that may be exacerbated during the pandemic, beyond the mitigation of sexual violence and abuse and other forms of GBV that are caused by the delivery of humanitarian aid. This can be achieved by ensuring that GBV services continue and by prioritizing and implementing community-based prevention interventions that address the root causes of violence [6, 17, 28–31].

Challenge unequal division of labor and harmful gender roles

- Address women’s unequal burden of domestic labor/household tasks and unpaid care work by first acknowledging the issue explicitly, and then redistributing responsibilities within families with support from government and employers (for example, parental leave policies and flexible work hours).
- Address the power imbalances that hinder women and non-binary people from making decisions in seeking health care during the global health crisis, and more generally, empower inclusion of people of diverse genders in decision-making.
- Campaign/promote efforts to challenge harmful stereotypes about women’s and men’s work that contribute to the gender imbalance of workers in the health sector, with roles considered “less prestigious” (such as nurses and community health workers) stereotyped as “female,” while leadership positions and doctors tend to be considered "male" domains.
- Address gender disparities in access to technology, literacy and education, including the social norms and practices such as child marriage that disproportionately impact girls.
- Prioritize social protection programs to support groups that may be adversely affected by an economic downturn, for example female-headed households.
- Include intersectional analysis and tangible solutions for groups of people who experience the impact of overlapping identities and power hierarchies such as sexuality, gender, (dis)ability, race, education status, economic status, immigration status, etc.

Guarantee access to health care

- Provide gender-sensitive support for frontline health workers, for instance personal protective equipment that are suitable for female bodies, menstrual hygiene management kits, or additional financial support.
- Ensure that sexual and reproductive health services and provision of information are not disrupted and explicitly provided with resources to allow for access and uptake.
- Ensure that mental health services are not disrupted but are available through alternative modalities such as online/virtual methods if in-person services are not possible. These should be regarded as essential health services, with attention paid to examining potential gender gaps or disparities in access to, uptake of, and efficacy of these services.

Engage and consult with women and youth (especially girls) groups meaningfully

- Include efforts to add more equal representation of women in leadership, for example within the COVID-19 taskforces.
- Ensure plans are developed with meaningful participation of women’s groups and networks, and feminist activists.

Mainstream gender in monitoring and evaluation

- Ensure that gender is embedded into all monitoring and evaluation indicators, and also as a stand-alone gender-specific indicator in monitoring and evaluation frameworks.

Minimize the gender digital divide

- Consult with local feminist organizations to understand the extent to which women, girls, and gender minorities may have reduced access to technology, digital tools, and digital literacy.
- Address context-specific barriers and harmful norms that affect the inequitable use of digital tools in order to maximize digitized service provision and data collection among women, girls, and gender minorities.

International organizations such as United Nations, international financial institutions such as the World Bank and International Monetary Fund, as well as international non-governmental organizations should:

- Support capacity strengthening of governments who need to collect and report on sex-disaggregated data.
- Develop best practices pertaining to the collection of gender-sensitive primary data using remote data collection tools.
- Intentionally engage government actors to normalize issues that are not yet widely understood in various contexts such as intersectionality and LGBTQI + inclusion.

## Conclusion

The COVID-19 pandemic magnified gender-based disparities faster than governments, multi-national organizations, and the humanitarian sector could respond. There is a dire need for adequate public health preparedness and response plans that not only address community transmission but integrate the rights, needs, and health of women, girls, and gender minorities in humanitarian settings. Our analysis shows that although most countries surpassed the bar of being “gender-blind” in their pandemic planning, gender has not yet been mainstreamed into policy planning. Further, no plans were considered “gender-transformative,” indicating that most consideration of gender remains superficial or remains siloed into issue areas that specifically affect women, such as GBV or reproductive services. Given the complex interplay between gender inequality and COVID-19 in humanitarian settings, national preparedness and response plans that are devoid of critical gender considerations have dire consequences: failure to safeguard the rights and health of women, girls, and non-binary persons, erosion of long-term gender transformation efforts such as the Women Peace and Security Agenda, and neglecting to address gendered chains of transmission, thereby incubating COVID-19 on a bedrock of structural gender inequity. More critical and intersectional approaches to crisis planning should be adopted in the future to mitigate the significant gender disparities exacerbated by significant societal disruptions, especially in the most fragile settings.

## Supplementary Information

**Additional file 1.** Supplementary - Indicators and Scores.xlsx/Full table of scored indicators.

## Data Availability

All data generated or analysed during this study are included in this published article [and its additional files].
